# Examining the association between menstrual symptoms and health-related quality of life among working women in Japan using the EQ-5D

**DOI:** 10.1186/s12905-021-01462-7

**Published:** 2021-09-07

**Authors:** Kyoko Shimamoto, Mana Hirano, Osamu Wada-Hiraike, Rei Goto, Yutaka Osuga

**Affiliations:** 1 Graduate School of Health Management, Keio University, 35 Shinanomachi, Shinjyuku, Tokyo, 160-8582 Japan; 2 Department of Obstetrics and Gynecology, Mitsui Memorial Hospital, Kanda-Izumi-cho 1, Chiyoda-ku, Tokyo, 101-8643 Japan; 3 Department of Obstetrics and Gynecology, Faculty of Medicine, University of Tokyo, 7-3-1 Hongo, Bunkyo-ku, Tokyo, 113-8655 Japan; 4 Graduate School of Business Administration, Keio University, 4-1-1 Hiyoshi, Yokohama, Kanagawa 223-8526 Japan

**Keywords:** Menstruation, Menstrual symptoms, Quality of life (QoL), Health related quality of life (HRQoL), Utility values, EQ-5D, EQ-5D-3L, Japan, Health economic evaluations

## Abstract

**Background:**

Menstrual symptoms have been identified as a substantial burden among women of reproductive age, affecting their health status and quality of life globally. A range of menstrual symptoms have been studied as they affect the health-related quality of life (HRQoL), showing variations across specific menstrual symptoms and study settings. A major concern is demonstrated due to menstrual symptoms in women’s professional and social life, and consequently societal and economic loss for women and the society at large. Yet evidence is scarce that estimates the index form HRQoL score related to menstrual symptoms that is needed for health economic evaluations.

**Methods:**

This study aims to investigate the association between menstrual symptoms and the HRQoL among working women in Japan in an index form, using a self-reporting questionnaire (n = 6048). The EQ-5D-3L (EuroQoL 5-dimension 3-level) is used that is a widely used tool to measure health outcomes for health economic evaluations globally. Multivariate regression analysis is conducted to assess the association between the HRQoL score and specific nineteen physical and mental conditions related to menstruation (e.g., pain, heavy bleeding, concentration, negative affect).

**Results:**

The index form HRQoL score for menstrual symptoms is estimated as 0.682 in the study population (where a score one suggests perfect health). The association of the HRQoL score varies substantially across the menstrual symptoms. Several of the physical conditions and disorders show a substantial negative association with the HRQoL score. Also, most of the mental and psychological issues are significantly and negatively related to the HRQoL score.

**Conclusions:**

This study suggests that HRQoL is substantially and negatively affected by menstruation among working women in Japan. Distinct variations of negative influences across menstrual symptoms underscore the multi-dimensional nature of menstruation and consequently the need of collective interventions to address these difficulties. The evidence of HRQoL continues to be an important area for future research on women’s health and health economic evaluations to inform effective and efficient resource allocations for relevant health policies and financing strategies.

## Background

Menstrual symptoms have been recognized as one of the major burdens among women of reproductive age globally, which negatively influence their health status and quality of life. A range of menstrual symptoms and relevant disorders have been studied in the literature including heavy menstrual bleeding (HMB or menorrhagia), pre-menstrual syndrome (PMS), primary dysmenorrhea (PD) and premenstrual dysphoric disorder (PMDD) among others [1–10]. The prevalence of these menstrual symptoms and disorders though has been substantially varied across the setting and characteristics of study populations in terms of age, social status (e.g., student, professional), comorbidities and past medical history for instance [11–14]. Yet existing literature consistently underscores a major concern and a significant negative impact of menstrual symptoms on the quality of life, suggesting negative impacts on their social and professional life and consequently the societal and economic loss [1–10].

Evidence generally suggests that menstrual symptoms and disorders are common among women of reproductive age. For example, a study from the UK demonstrated concerns and perceptions of women age 25–44 (n = 906), particularly menstrual pains as a major concern (27%), while the judgement as a problem was contingent on social circumstances surrounding respondents (e.g., employment status, type of work, and support received) [11]. An Australian study among women age 20–39 (n = 1575) found that the half (49%) experienced menstrual pains (moderate, severe or very severe), while around one third (28%) reported HMB [13]. In Asia, a survey employing a national level sampling in Japan (n = 19,254) revealed that the majority of women of reproductive age 15–49 suffered from menstrual symptoms (74%) and menstrual pains (50%) [12]. Further, a national-level study in Taiwan among medical professionals (n = 7653) showed variations of the prevalence of menstrual disorders and health care seeking behaviors across medical professions [15].

Besides the prevalence of menstrual symptoms, another set of studies investigated the quality of life (QoL) or health-related quality of life (HRQoL) associated with menstrual symptoms. The SF-36 (the 36-Item Short Form Health Survey) has been the most commonly used tool involving the defined eight dimensions (e.g., physical function, social functioning, mental health, general health, energy/fatigue, pain) [16].

Most of these studies focused on menorrhagia or heavy menstrual bleeding (HMB) in estimating its negative impact on the HRQoL. For example, a systemic review affirmed the negative impact of menorrhagia on the HRQoL among women in general and those with inherited bleeding disorders [7]. Further, menorrhagia (or HMB) and HRQoL were assessed using the SF-36 in several countries including Pakistan, Sweden and Turkey [2–4], while in the US, HMB was assessed using the Menorrhagia Impact Questionnaire (MIQ) [5]. These studies suggest that menorrhagia (or HMB) is generally associated with a significantly lower HRQoL score, with variations by dimension and study setting [2–4, 7]. Furthermore, evidence also exists that focused on other menstrual symptoms using the SF-36. Studies in Turkey compared HRQoL scores among women in different groups, showing disproportionate influences by menstrual symptom (e.g., Premenstrual Dysphoric Disorder, Premenstrual Symptoms, dysmenorrhea) [1, 9]. Menstrual and menarche experience was assessed among the nationally representative healthy women age 18–40 in Iceland, finding their negative influence on the mental and physical health [8].

However, limited studies assessed the HRQoL using an index form. The index form HRQoL score, also called as a utility value, is a single cardinal value assigning 0.0 for death and 1.0 for perfect health [17, 18], which is needed to calculate Quality Adjusted Life Years (QALYs). In Hungry, the utility value was estimated for severe and mild hypothetical Primary Dysmenorrhea (PD), finding the mean utility values as 0.85 for the severe PD and 0.94 for the mild PD [6]. Also, premenstrual dysphoric disorder (PMDD) was assessed in Japan using the EQ-5D-3L (EuroQoL 5 dimensions 3 levels), showing utility values as low as 0.409 during the premenstrual period and 0.795 as a mean value for all menstrual cycles [10].

Evidence of menstrual symptoms and relevant HRQoL is still limited globally, yet there are studies that shed light on the HRQoL and relevant societal and economic burdens due to menstrual symptoms in Japan. A total annual economic burden due to menstrual symptoms among Japanese women was estimated as 8.6 billion USD, of which work productivity loss accounted for 72% of the burden (n = 19,254) [12]. Also, Miyauchi [19, 20] studied negative impacts of menstrual symptoms among working women, finding concerns in terms of women’s health status, hospital visits, admission, discharge and rejoining office.

These studies consistently suggest that menstrual symptoms substantially and negatively impact women’s quality of life and the society. Although the index form HRQoL score could be estimated based on the commonly used tools such as the SF-36 and SF-12 [21–24], the evidence of the index form HRQoL score related to menstrual symptoms is still scarce [6, 10]. Due to increasing attentions to economic evaluations such as Health Technology Assessment (HTA) and cost-effectiveness analysis globally, the need of the index form HRQoL score (0–1 scale) has been also underscored for the major symptoms and disorders affecting the population.

Further, evidence of HRQoL surrounding menstruation is still limited to heavy menstruation bleeding (HMB) or menorrhagia [2–5, 7], and the HRQoL related to other menstrual symptoms are less studied. Consequently, such comparisons of various menstrual symptoms are scarce, while each symptom may influence the HRQoL to different extents. Due to the substantial burden that these women carry, it is increasingly important to compare and identify the extent of major menstrual symptoms as they impose barriers to women’s quality life and work.

This study thus aims to examine the menstrual symptoms as they influence the quality of life among Japanese women, particularly the index form HRQoL score. The relationship of the HRQoL score with each menstrual symptom is assessed that suggests the potential, disproportionate influence of each symptom on the HRQoL.

## Methods

### Data

A self-reporting questionnaire survey was conducted about menstrual symptoms among women of reproductive age. Data were collected from November 2016 to January 2017 using a convenience sampling approach targeting major commercial companies from which a consent was obtained at the company level (e.g., consumer goods manufacturer, insurance, pharmaceutical, and transportation – in total four companies). A survey was administered by a research company (INTAGE Inc.) using a mixed approach – an internet base and a paper base as managed differently by the company.

For this analysis, data are derived particularly from women who have menstruation, do not take any hormone drug, and whose data are available to calculate the HRQoL score. These criteria consider that women using hormone drug may experience menstrual symptoms less than those who do not use medications and/or may be more likely to receive relevant treatment thus report higher quality of life. The analytic sample for this study is 6048 women of reproductive age who work for a company.

This study used a generic preference-based measure to assess the health-related quality of life (HRQoL) score as described later in detail (under the subsection of measures). Respondents were asked to complete the self-reporting questionnaire about their menstrual and pre-menstrual experience in the preceding three months at the time of the survey, with the definition of pre-menstrual period as three to ten days before the onset of menstruation in this survey.

This study was conducted in Japan where previous studies consistently reported that the majority of women suffer from menstrual symptoms and/or relevant disorders and experience difficulties in seeking necessary medical care and pursuing professional work [12, 19, 20, 25]. In Japan, cancer has been the leading cause of death among both men and women, accounting for around a quarter of deaths among women [26]. Among females, the incidence rates of reproductive cancers (breast and uterus) rank at one of the highest among other cancers (e.g., colon, stomach, lung) [27]. The majority of women reaching around three-quarters (74%) experience menstrual symptoms as reported from the survey at the national level [12].

### Measures

This survey comprised topics and questions related to demographics, menstrual symptoms and relevant heath service use. In particular, the questionnaire asked about demographics such as age and employment (e.g., profession, work modality); pregnancy and delivery experience; reproductive health service use and relevant expense; menstrual symptoms in terms of timing (i.e., regular or irregular menstruation), duration, characteristics and their impacts on professional work and general life.

The questionnaire development was coordinated by one of the authors and a sub-committee of the Japan Society of Obstetrics and Gynecology (JSOG). Specific questions were designed based on clinical consultations with patients in the Japanese context; consolidated questions were reviewed and validated by the sub-committee of the JSOG, involving pre-testing with a small group of women of reproductive age. The list of menstrual symptoms in the questionnaire comprised nineteen physical and mental conditions, surrounding pain, concentration, autonomic reactions, water retention and negative affect – that are related to the dimensions defined by the existing relevant tool [28]. Women were asked about the experience of these nineteen menstrual symptoms and also identified the specific symptoms that substantially affected professional work and general life respectively.

This survey employed the EQ-5D-3L (The EuroQol 5-dimension 3-level) which has been a widely used tool in clinical trials and public health studies, as validated and employed in numerous settings and recommended particularly for health technology assessments as a health outcome measure. This tool allows to rate respondents’ health status in the following five dimensions: mobility, self-care, usual activities, pain/discomfort, and anxiety/depression. Among the available versions of the EQ-5D (i.e., EQ-5D-3L and EQ-5D-5L for adults), this study used the EQ-5D-3L which assesses each dimension in three levels of severity in the answer option (e.g., “no problem”, “some problems”, or “unable”) [29]. In this study, a suboptimal version of the EQ-5D-3L translated in Japanese was employed, and the respondents were asked to rate their health status at the time when menstrual symptoms were at the worst state.

### Analytic steps

Analysis was conducted in the following steps. First, descriptive analysis was conducted, showing the respondents’ demographic characteristics, general reproductive health experience and the prevalence of the selected menstrual symptoms. Second, the HRQoL score was calculated. According to the EQ-5D-3L tool, the respondents indicated their health state among the three levels, thus one-digit number was assigned for each of the five dimensions (with the answer range 1–3). The digits for each dimension were combined into a five-digit number (e.g., 11111). The estimation of the HRQoL score was based on the validated valuation method in the Japanese context [30]. For example, the respondent who answered “no problem” for all five dimensions resulted in the five-digit number of “11111”, which was translated into the HRQoL score of 1.000 as perfect health. For the respondents who answered “unable”, “extreme pain” or “extremely anxious” with the five dimensions (i.e., 33333), the HRQoL score was estimated as—0.111. The HRQoL score was estimated for each of the combined, different five-digit number, resulting in two-hundred and forty-three different health states (i.e., 3^5^ = 243).

After the estimation of the HRQoL score, the association of the HRQoL score with each menstrual symptom was assessed respectively. Multivariate linear regression was employed, regressing the HRQoL score on the nineteen menstrual symptoms (plus a category as “other”) and the regularity of menstruation cycle, in which the respondent’s age was included as a control variable.

## Results

### Descriptive statistics

Table 1 shows the descriptive statistics of the respondent’s demographics and the general experience with reproductive health and menstruation. The respondents’ age ranges through women’s reproductive age, from teenage to early fifty. Almost all the respondent works on a full-time contract (97.2%). One-third of the women reported to have delivery and maternity experience (30.0%), and around one in four of these women have irregular menstruation (25.8%).

**Table 1:** Descriptive analysis of the study sample women (n = 6048)

Variables	Frequency	Proportion	SE
*Demographics and general reproductive health*			
Age			
Under age 20	8	0.13	0.000
Age 20–29	1724	28.62	0.006
Age 30–39	1806	29.98	0.006
Age 40–49	2108	34.99	0.006
Age 50 and over	378	6.27	0.003
Employment status			
Full time (daytime)	3844	63.99	0.006
Full time (including night time)	1827	30.41	0.006
Full time (short time)	166	2.76	0.002
Part time	170	2.83	0.002
Delivery and maternity experience under employment			
Yes	1502	30.04	0.006
No	3498	69.96	0.006
Regularity of menstrual cycle			
Yes	4490	74.24	0.006
No	1558	25.76	0.006
*Menstrual symptoms (multiple options)*			
Abdominal pain	4241	70.61	0.006
Back ache	3092	51.48	0.006
Sleepiness	2895	48.2	0.006
Diarrhoea and/or constipation	2578	42.92	0.006
Irritation	2514	41.86	0.006
Breast swelling	2419	40.28	0.006
Fatigue	2106	35.06	0.006
Headache	2016	33.57	0.006
Depressed mood	1838	30.6	0.006
Appetite increase/decrease	1751	29.15	0.006
Swelling	1311	21.83	0.005
Heavy bleeding	1202	20.01	0.005
Shoulder pain	1152	19.18	0.005
Concentration loss	1143	19.03	0.005
Lethargy	1025	17.07	0.005
Coldness	780	12.99	0.004
Itching	466	7.76	0.003
Hot flash	326	5.43	0.003
Sweating	244	4.06	0.003
Other	208	3.46	0.002

Also, Table 1 shows the experience of the selected nineteen menstrual symptoms plus a category as “other” with multiple choices. Over two-thirds of the respondents experience abdominal pains (70.6%), and around half reported backache (51.5%) and sleepiness (48.2%). Besides, around two in five women experience diarrhea and/or constipation (42.9%), irritation (41.9%), breast swelling (40.3%); around one-third of women experience fatigue (35.1%), headache (33.6%) and depressed mood (30.6%).

### Health-related Quality of Life (HRQoL) scores and multivariate analyses

The HRQoL score for menstrual symptoms is estimated as 0.682 in the study population, as calculated as a mean of the HRQoL score among the study population. Also, Table 2 shows the result of the multivariate analysis, regressing the HRQoL score on the selected menstrual symptoms and the regularity of menstruation cycle controlling for age.

**Table 2:** Multivariate linear regression analysis of the Health-related Quality of Life (HRQoL) score on menstrual symptoms (n = 5609)

	Coefficient (b)	*p* value	Confidence Interval	
Independent variables—Menstrual symptoms				
Abdominal pain	− 0.103	0.000	− 0.112	− 0.093
Back ache	− 0.036	0.000	− 0.045	− 0.026
Sleepiness	− 0.013	0.009	− 0.022	− 0.003
Diarrhoea and/or constipation	− 0.028	0.000	− 0.038	− 0.019
Irritation	− 0.005	0.308	− 0.015	0.005
Breast swelling	− 0.004	0.428	− 0.013	0.006
Fatigue	− 0.032	0.000	− 0.042	− 0.021
Headache	− 0.046	0.000	− 0.056	− 0.035
Depressed mood	− 0.045	0.000	− 0.057	− 0.034
Appetite increase/decrease	− 0.001	0.828	− 0.012	0.010
Swelling	− 0.002	0.798	− 0.014	0.011
Heavy bleeding	− 0.061	0.000	− 0.074	− 0.049
Shoulder pain	0.008	0.208	− 0.005	0.022
Concentration loss	− 0.035	0.000	− 0.049	− 0.022
Lethargy	− 0.038	0.000	− 0.053	− 0.023
Coldness	− 0.023	0.005	− 0.039	− 0.007
Itching	0.007	0.428	− 0.010	0.025
Hot flash	− 0.017	0.221	− 0.043	0.010
Sweating	− 0.014	0.379	− 0.045	0.017
Other	− 0.067	0.000	− 0.098	− 0.037
Control variables				
Regularity of menstruation	0.020	0.000	0.009	0.032
Age 20–29	− 0.037	0.549	− 0.157	0.084
Age 30–39	− 0.037	0.549	− 0.157	0.083
Age 40–49	− 0.022	0.715	− 0.143	0.098
Age 50 and over				
Intercept	0.877	0.000	0.757	0.998
Model statistics				
F test	69.070	0.000		
R-squared	0.254			
Root MSE	0.174			

Among the selected nineteen specific menstrual symptoms, there are eleven symptoms that are negatively related to the HRQoL score with statistical significance (*p* < 0.05). Lower abdominal pains have marked a substantial negative association (b = -0.103), followed by heavy menstruation bleeding (b = -0.061), headache (b = -0.046) and depressed mood (b = -0.045). Other symptoms that have been significantly and negatively related to the HRQoL score include lethargy (b = -0.038), backache (b = -0.036), concentration loss (b = -0.035), fatigue (b = -0.032), diarrhea and/or constipation (b = -0.028), coldness (b = -0.023) and sleepiness (b = -0.013). Remaining eight symptoms do not show a statistically significant association with the HRQoL score, while the category of “other symptoms” also shows a significant negative relationship (b = -0.067).

Moreover, regularity of menstruation is positively and significantly associated with the HRQoL score (b = 0.020). Respondents’ age on the other hand does not show a statistically significant relationship.

## Discussion

This study has examined the range of menstrual symptoms and compared their association with the quality of life among working women in Japan. The results from this analysis suggests that particular menstrual symptoms substantially and negatively affect the quality of life and consequently societal and economic loss, relative to other symptoms, as estimated in terms of the index form HRQoL score.

The most influential menstrual symptoms are physical pains and disorders including abdominal pain, heavy bleeding (menorrhagia) and headache among the study population. This key result is consistent with previous studies on menstrual symptoms, many of which have underscored the substantial burden of heavy bleeding and physical pains and consequently the deteriorated health and wellbeing among women in Asia, Europe, Oceania and the United States [1–10]. Although the comparison of menstrual symptoms is limited in the exiting literature, this study suggests the substantial relative burden of these physical pains and disorders over other menstrual symptoms.

Further, the result from this study highlights that Japanese women also experience significant difficulties in terms of mental and psychological wellbeing due to menstruation. Indeed the second set of menstrual symptoms that are significantly and negatively related to the HRQoL score has comprised depressed mood, lethargy and concentration loss. These symptoms may also deteriorate work productivity and lead to the major societal and economic loss. Whilst some of the previous studies have reported these mental and psychological aspects [2–4, 6, 7, 9], this evidence suggests the significance of these aspects to be further addressed in future research and interventions.

Moreover, the positive relationship between the HRQoL and the regularity of menstruation cycle underscores the importance for women to seek relevant, timely health care and support when experiencing irregular menstruation. Besides the handful of studies on the prevalence of irregular menstruation and its negative relationship with health status, well-being and work productivity [31–35], evidence from this analysis cautions against the underestimation of its negative impact.

This study has generated evidence of quality of life, in particular the index form HRQoL score related to menstrual symptoms. As such this evidence enables indicative comparisons of the burden of menstrual symptoms relative to other common symptoms, disorders and/or diseases among women of reproductive age. For example, besides the evidence of HRQoL score from Japan and Hungry related to primary dysmenorrhea (PD) and premenstrual dysphoric disorder (PMDD) [6, 10], a recent Japanese study on the recovery after laparoscopic myomectomy among patients with uterine fibroids estimated the HRQoL score from 0.58 to 0.97 (for the different clinical states) [36]. In Japan, evidence of breast cancer also demonstrates a range of HRQoL score at different clinical stages, for example 0.836 prior to the first chemotherapy and 0.749 prior to the second chemotherapy [37]. Thus the comparison of the index form HRQoL score suggests that the utility value of menstrual symptom is not negligible, even though the periodic and temporal nature of menstrual symptoms leads to different implications from these clinical states.

As such, this study entails strengths to advance future research, women’s health status and quality of life. Evidence of the index form HRQoL score is important as is required to undertake economic evaluation for estimating cost effectiveness – Incremental Cost Effectiveness Ratios (ICERs) of reproductive health interventions. Indeed previous studies have suggested that girls and women tend to delay and/or avoid visiting gynecologists, even though they experience menstrual symptoms in Japan and elsewhere [19, 20, 25, 38, 39]. Given this challenge under constrained support in office, this evidence may have a potential to facilitate discussions towards promoting measures and enabling environment for such working women to seek relevant care at the appropriate timing. Due to the scarcity of evidence of the index form HRQoL score related to menstrual symptoms globally, this evidence may also be a useful data point elsewhere and facilitate research in other settings.

This study though also entails several limitations. First, this survey employed a convenient sampling approach, which was not a nationally representative. Second, the calculation of the HRQoL score employed a complete case analysis. Third, the survey questionnaire asked the health state of respondent at the time when menstrual symptoms were most severe, thus the HRQoL score during the overall menstrual period may have been underestimated. Fourth, this questionnaire was developed based on the expert’s opinion and clinical practice in the Japanese context. Existing international tools such as the Menstrual Distress Questionnaire could have been adopted or adapted in the present study [28]. Beyond the selected nineteen menstrual symptoms, there may have been other specific symptoms associated with the HRQoL. Fifth, multivariate analysis could have included additional control variables besides age if such data were collected. Last, this survey used a suboptimal version of the EQ-5D-3L. Future studies should address these limitations, and they could also compare women’s quality of life across different groups (e.g., those who use hormonal treatment and/or other treatment) in exploring the intervention approach for promoting women’s gynecology visits in Japan and elsewhere.

## Conclusions

In summary, this study suggests that the HRQoL is substantially and negatively affected by menstrual symptoms among working women in Japan, who experience significant challenges in their professional and social life. Distinct variations of negative influences across menstrual symptoms underscore the multi-dimensional nature of menstruation and consequently the need of collective interventions to address these difficulties. The evidence of HRQoL continues to be an important area for future research on women’s health and health economic evaluations to inform effective and efficient resource allocations for relevant health policies and financing strategies.

## Data Availability

The datasets generated and analysed during the current study are not publicly available due to the contract with the participating company in terms of privacy but are available from the corresponding author on reasonable request.

## Abbreviations

EQ-5D: The EuroQol 5-dimension
EQ-5D-3L: The EuroQol 5-dimension 3-level
HRQoL: Health-related quality of life
